# The governance of land use strategies: Institutional and social dimensions of land sparing and land sharing

**DOI:** 10.1111/conl.12429

**Published:** 2017-12-13

**Authors:** Tolera S. Jiren, Ine Dorresteijn, Jannik Schultner, Joern Fischer

**Affiliations:** ^1^ Faculty of Sustainability Leuphana University Lueneburg Scharnhorststrasse 1 21335 Lueneburg Germany

**Keywords:** biodiversity, conservation, food security, governance, institutions, intensification, land sharing, land sparing, land use strategy

## Abstract

Agricultural land use is a key interface between the goals of ensuring food security and protecting biodiversity. “Land sparing” supports intensive agriculture to save land for conservation, whereas “land sharing” integrates production and conservation on the same land. The framing around sparing versus sharing has been extensively debated. Here, we focused on a frequently missing yet crucial component, namely the governance dimension. Through a case‐study in Ethiopia, we uncovered stakeholder preferences for sparing versus sharing, the underlying rationale, and implementation capacity challenges. Policy stakeholders preferred sparing whereas implementation stakeholders preferred sharing, which aligned with existing informal institutions. Implementation of both strategies was limited by social, biophysical, and institutional factors. Land use policies need to account for both ecological patterns and social context. The findings from simple analytical frameworks (e.g., sparing vs. sharing) therefore need to be interpreted carefully, and in a social‐ecological context, to generate meaningful recommendations for conservation practice.

## 1 INTRODUCTION

Improving food security and biodiversity conservation are two prominent goals for sustainability. Food security refers to the stable supply of accessible, nutritional, culturally acceptable food (FAO, 2014), while biodiversity is the variability among organisms and ecosystems (Convention on Biological Diversity, 1992). Harmonizing food security and conservation is important (Tscharntke *et al*., 2012), but can be challenging because of pressures such as population growth, land scarcity, and climate change (Godfray *et al*., 2012). The identification of appropriate land use strategies could be one way to facilitate improved integration of food security and conservation (Macchi, Grau, Zelaya, & Marinaro, 2013).

To this end, a prominent framework distinguishes between “land sparing” and “land sharing” (Balmford, Green, & Scharlemann, 2005; Green, Cornell, Scharlemann, & Balmford, 2005). Land sparing implies the spatial segregation of production and conservation (Fischer *et al*., 2008; Grau, Kuemmerle, & Macchi, 2013). It is based on the recognition that agricultural area expansion is a critical threat to biodiversity (Balmford *et al*., 2005), and therefore supports the creation of protected areas, while allowing for production zones to be intensified (Fischer *et al*., 2008). In contrast, land sharing denotes production and conservation taking place on the same land, using biodiversity‐friendly methods (Green *et al*., 2005).

The sparing versus sharing framework has been widely used – for instance, in relation to the conservation of birds and plants (Phalan, Onial, Balmford, & Green, 2011; Egan & Mortensen, 2012), coffee management (Chandler *et al*., 2013; Aerts *et al*., 2017) and local livelihoods (Dressler, de Koning, Montefrio, & Firn, 2016). However, debate is ongoing about its applicability to real‐world problems. Among others (Fischer *et al*., 2014), criticisms include the possible oversimplification of complex systems, and limited consideration of social and governance dimensions, including institutions and stakeholder preferences (Chandler *et al*., 2013; Kremen, 2015). Perhaps most importantly, the link between agricultural intensification and the creation of protected areas may be weak or absent (Phalan *et al*., 2011; Phelps, Carrasco, Webb, Koh, & Pascual, 2013), such that agricultural intensification could even exacerbate agricultural expansion. This may occur in the case of the “Jevons paradox,” where improved land use efficiency creates incentives for the further expansion of intensive land use (Matson & Vitousek, 2006; Desquilbet, Dorin, & Couvet, 2016).

Here, we investigated governance dimensions of the sparing versus sharing framework in a multilevel governance context. We focused on southwestern Ethiopia, an internationally recognized biodiversity hotspot (Tadesse, Zavaleta, Shennan, & FitzSimmons, 2014) that has experienced major declines in forest cover (Ango, Börjeson, Senbeta, & Hylander, 2014), and has low food security by international standards (Oromia Bureau of Finance and Economic Development, 2012). Our aims were to: (1) elicit the preferences for sparing versus sharing by different stakeholders involved in food security and biodiversity conservation, from local community to national government; (2) understand the justifications for these different preferences; and (3) explore capacity limitations in the implementation of both land sparing and land sharing. We contextualize our findings by comparing them with studies from other parts of the world. We argue that social and governance dimensions should be more routinely considered in discussions about land sparing versus land sharing.

## 2 METHODS

### 2.1 Study area

The study was conducted in Oromia regional state, Jimma zone, between October 2015 and February 2016. Ethiopia consists of nine regional states, which are demarcated on the basis of linguistics and ethnic lines (see supplementary material). The country has five administrative levels: the federal, regional, zone, woreda (district), and kebele (municipality) levels. Within Jimma zone, we selected three woredas (Gumay, Gera, and Setema), and two kebeles within each of these. The selected six kebeles (Kuda Kufi, Berwerengo, Kela Hareri, Borcho Deka, Gido Bere, Difo Mani) varied in forest cover and altitude, which are important ecological and socioeconomic drivers. We engaged with stakeholders at all five formal levels of governance.

Stakeholders are organizations and community groups who affect or are affected by decisions in a specific context (Reed *et al*., 2009). We identified relevant stakeholders – those involved in the governance of food security, biodiversity conservation, or both – through snowball sampling. We broadly conceptualized food security and involved production‐related stakeholders including farmers and agricultural offices; access‐related stakeholders such as financial institutions; utilization‐related stakeholders such as health offices; and stability‐related stakeholders such as administration offices (Table S1). For biodiversity, we involved stakeholders engaged with forest, wildlife, and other biodiversity conservation aspects (Table S1).

We used a bottom‐up process of stakeholder identification, starting with farming communities in each kebele. To avoid bias, we involved a diversity of stakeholders in terms of wealth, gender, and household location (Table S2). Farmers were categorized into rich versus poor, following an official wealth classification (see supplementary material). Community‐level discussants were identified through the help of local guides (see supplementary material), considering their level of knowledge and experience, ability to articulate opinions, and willingness to participate.

During our work in the communities, we asked farmers to identify stakeholders they work within the context of food security or biodiversity conservation, both horizontally (i.e., within the kebele) and vertically (i.e., at higher levels). We followed this procedure to identify stakeholders up to the federal level. In total, we identified 244 stakeholders. Eighty of these were directly involved in land use policy or implementation strategies, and these form the basis for this article (Table S2). The remaining stakeholders were also involved in food security and/or biodiversity governance, but devising specific land use policies or implementing specific management decisions was not part of their organizational mandates (see supplementary material). For government organizations, we interviewed relevant representatives, including chairpersons, deputies, senior personnel, and technical experts.

### 2.2 Data collection and analysis

We collected data using semistructured interviews and (at the community level) focus group discussions. Both were guided by three themes: (1) identification of preferences concerning land use strategies (i.e., land sharing, sparing, or a combination); (2) justification of these preferences; and (3) capacity limitations for the implementation of the preferred strategy. Before the actual study, we tested and refined our questions. Because the sparing/sharing terminology was unknown to stakeholders and to ensure a common understanding, we initially explained these concepts. We described land sparing as the separation of biodiversity conservation in protected areas and intensive agricultural land use outside protected areas; whereas land sharing was described as the integration of conservation and production on the same land. To assist stakeholders in understanding land sharing, we explained it using examples from the study area. First, sharing could be on the farmland, for example in the case of trees being grown in pastures or cropland. Second, sharing could also be in the forest, where traditional semiforest coffee production takes place (Aerts *et al*., 2017, Table S1). Interviews and discussions lasted for approximately 1 hour, and were documented using notes and voice recordings.

For analysis, we transcribed all 80 recordings and used content analysis in the software NVivo version 11. Here, we created three separate nodes for land sparing, land sharing, and mixed strategies; classified stakeholders according to their preferences of sparing, sharing, or a combination; and identified their responsibilities in policy‐making versus implementation. We then inductively created subnodes describing arguments related to the justification of preferred strategies and capacity limitations.

## 3 RESULTS

### 3.1 Aim 1: land use preferences

The preference regarding land use varied between stakeholders based on sector and wealth. Preferences included a “mixed strategy,” which favored sharing and sparing within the same landscape. For example, stakeholders may have argued for using external inputs such as agrochemicals in the farmland, but also argued for the maintenance of native trees in both the forest and throughout the farmland. Both land sharing and sparing were widely supported, with land sharing preferred (40% of 80 stakeholders), followed by land sparing (34%), and a mixture of both (26%).

**Figure 1:**
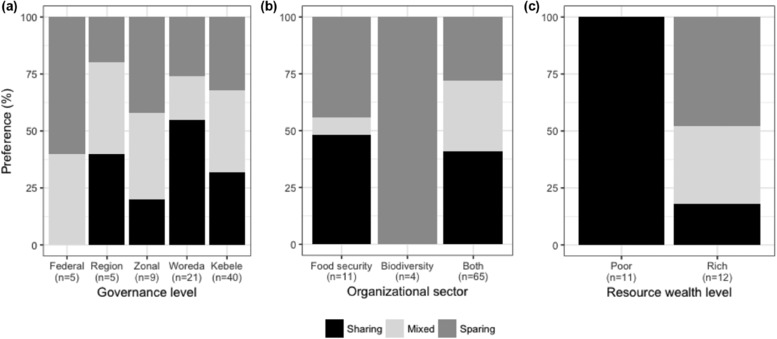
Land use preferences according to (a) level of governance, where federal to zone represents the policy‐making levels and woreda and kebele represent the implementation levels; (b) stakeholders' engagement in the governance of food security, biodiversity conservation, or both sectors; and (c) the wealth category of focus groups at the community level

Three key findings emerged. First, classifying the stakeholders according to policy‐making (zone, region, federal level) versus implementation levels (woreda, kebele), we found that land sharing was more popular at the implementation level, whereas land sparing and a mixture were preferred at the policy‐making level. At the implementation level, 45% and 23% of stakeholders preferred land sharing and a mixed strategy, respectively (*n* = 62), whereas at the policy level, land sparing and mixed‐land use strategies were preferred each by 39%, and land sharing was preferred by only 22% (*n* = 18, Figure 1a).

Second, stakeholders in the biodiversity sector usually preferred land sparing, whereas those in the food security sector preferred land sharing or a mixture. Of the 80 stakeholders interviewed, 14%, 5%, and 81% were involved in the governance of food security, biodiversity, or both, respectively. We found that 43% of stakeholders involved in both sectors preferred land sharing, while 29% preferred land sparing (*n* = 65). All biodiversity sector stakeholders preferred land sparing (*n* = 4, Figure 1b).

Third, a difference emerged at the community level between wealth categories. Poor community members unanimously preferred land sharing (100%, *n* = 11 groups of poor people). Half of the rich community stakeholders, in contrast, preferred land sparing (50%), followed by a mixed land use strategy (33%, *n* = 12, Figure 1c).

### 3.2 Aim 2: reasons underlying land use preferences

Preferences of land use strategies were determined by various factors (Table 1). Efficiency optimization was a prime justification for land sparing. In addition, all stakeholders with a preference for land sparing indicated that the conservation of dwindling forest biodiversity was a key motivation. Formal institutional support by the government for agricultural intensification (including access to inorganic fertilizers, pesticides, and improved seeds), and external factors such as population growth were other justifications for land sparing. For example, an interviewee from the agricultural sector explained that “the only viable solution in the face of climate change, population increase and land degradation is to use production enhancing technologies and increase yield.” An interviewee from the conservation sector stated: “Agricultural expansion and illegal settlement were primary causes of forest decline in the zone. Therefore, we [his organization] segregate agricultural land from conservation land, and demarcate [clear] conservation boundaries.”

**Table 1:** Justification given by stakeholders for their preference of land sparing versus land sharing, including proportion of respondents. For example, all 27 (100%) stakeholders preferring land sparing argued this strategy was best for biodiversity conservation

Preference	Justification	(%)
Land sparing (*n* = 27)	Best for biodiversity conservation.	100
	Good to increase yields via agricultural intensification.	89
	Land sparing has formal institutional support through government policy, strategy, and plans.	78
	There is good access to agricultural technology for intensification.	70
	There is an increase in population and demand for food.	52
	There are possible gains from forest conservation through emerging carbon markets.	41
	Land use specialization is better.	33
	Land sharing will not work to feed the population.	9
	Clear separation of land uses reduces conflict between stakeholders.	8
Land sharing (*n* = 32)	Land sharing is consistent with traditions and local institutional support: cultural relevance, traditional farming knowledge, ancestral human‐nature connections.	56
	Land sharing is preferable for cost‐benefit considerations: livelihood benefits of farm diversification outweigh the high costs of intensification (e.g., fertilizer).	56
	Land sharing is consistent with biophysical constraints and existing production systems: settlement structure, landscape and land ownership fragmentation, widespread shade coffee production.	41
	Resource conservation: land sharing is important for the conservation of forest and farm biodiversity.	31

In contrast, land sharing was commonly justified through the local importance of integrated landscapes. Both local institutional support and livelihood diversification were mentioned to justify the preference for land sharing (Table 1). Land sharing was supported by traditions and local institutions, and was related to cultural significance, farming traditions and knowledge, and ancestral experience and valuation of nature. A focus group member exemplified this by stating that “trees such as the sycamore fig [*Ficus sycomorus*], which is rare in the forest but occurs on farmland, provide shade under which conflicts are resolved, powers are transferred, oaths are made, and traditional cultural ceremonies are undertaken. We therefore prefer a sharing approach.” Cost‐benefit considerations also motivated a land sharing approach (Table 1). Most notably, livelihood diversification – having multiple sources of income to reduce risk – was considered an advantage of integrated landscapes. A poor female discussant explained this: “We produce varieties of crops in our small plots of land because we want to diversify our meals, and reduce the burden of crop failure.”

Second, high input costs explained preferences for land sharing. A focus group discussant explained: “We are forced to use fertilizer against our will. The added value to our produce through fertilizer use is lower than the cost of the fertilizer, and we have to sell assets to repay the cost of fertilizer.” Socioeconomic and biophysical landscape conditions were also considered. For instance, dispersed settlements, fragmented agricultural land holdings, and the widespread practice of shade coffee production were mentioned as reasons for preferring land sharing.

The strict protection of valuable trees in the forest, while implementing land sharing within the farmland, was the primary justification of stakeholders who preferred a mixed land use strategy (*n* = 21).

### 3.3 Aim 3: capacity limitations

The implementation of land sparing was perceived to be hampered by community attributes, limited organizational capacity, and resource limitations (Table 2). Community attributes included reluctance to adopt agricultural technologies such as agrochemicals and improved seeds. Examples of capacity limitation were a lack of technical knowledge, inability to enforce agricultural intensification, and insufficient finances. Moreover, coordination challenges between stakeholders in food and biodiversity, or contradictory plans and activities, were mentioned as significant constraints. One government employee explained that “we distribute honey production technologies, while the agricultural office is fostering the use of herbicides and fertilizers that harm bee colonies.” Similarly, a focus group participant stated that “development agents advise us to intensify the farmland while others such as cooperatives and unions provide us with seedlings to expand farm forestry and reduce the pressure on forests.”

**Table 2:** Capacity limitations for effective implementation of the preferred land use strategies, including proportion of stakeholders for different arguments. For example, out of the 27 stakeholders supporting land sparing, 21 (78%) described that community attributes were limiting capacity for the implementation of land sparing

Land use strategy	Capacity limitations	(%)
Land sparing (*n* = 27)	Community attributes: community is unwilling to adopt agricultural intensification.	78
	Capacity limitation in implementation: lack of coordination, and contradiction of sectoral plans, strategies and activities.	21
	Resource factors: limitations in skill and materials.	18
	Conflicting interests: the interest of the government and the community are not compatible. Promoted government services and technology are incompatible with local conditions.	9
	Farming system: agricultural land holdings are small and fragmented, and “shared” forest coffee is widespread.	4
	Governmental problems: there are structural fluctuations in offices and responsibilities, and administrative inconsistency between offices.	3
Land sharing (*n* = 32)	Imposition of technologies, strategies, and plans does not match the needs and capabilities of the community.	14

Implementation challenges of land sharing focused chiefly on incompatibilities between community and government stakeholders. The forced imposition of agricultural technologies was perceived to impede the traditional continuation of land sharing (Table 2). One development agent stated that “our services are not in line with the community we ought to serve. However, we keep doing it as long as we are directed to do so from our administration.”

## 4 DISCUSSION

This study revealed previously underexplored governance challenges for the implementation of land sparing or land sharing. Although both food security and biodiversity conservation are prominent goals in our study area, we identified institutional and social challenges to their integration. As we discuss below, similar challenges are likely to apply to other smallholder farming landscapes around the world.

### 4.1 Preferred land use strategies differ between stakeholders

Stakeholders differed in their views how to best harmonize food security and biodiversity conservation. Importantly, preferences for land use strategies were not limited to a dichotomous distinction of strategies into “sparing” versus “sharing” but often recognized the benefits of a mixed strategy. This empirical finding is consistent with previous arguments that a combination of strategies – adjusted to local conditions – is often required (Fischer *et al*., 2008; Kremen, 2015). It also confirms the notion that land sparing and sharing is an insufficiently nuanced framing of local realities (Kremen, 2015; Dressler *et al*., 2016). At worst, the oversimplification of complex realities could impede rather than foster the harmonization of food production and biodiversity conservation (Butsic, Baumann, Shortland, Walker, & Kuemmerle, 2015). For instance, empirical findings by Habel *et al*. (2015) in Kenya and Law *et al*. (2015) in Indonesia indicated that land use policy involves complex and integrated decisions, highlighting that the simple implementation of either land sparing or land sharing would generate suboptimal outcomes for both food security and biodiversity conservation.

Preferences for land use strategies differed across governance levels and sectors. Locally, although there was no difference on the preference of land use strategies between the six kebeles, we found an important difference between poor and rich farmers. Poor farmers clearly preferred land sharing, whereas rich farmers – who can afford agrochemicals and may produce surplus for markets – more often favored land sparing. Whereas rich farmers may seek to maximize yields through commercialized farming, poor farmers may seek to ensure basic household needs, minimize risks, and maximize livelihood resilience against shocks. This finding is in line with research from Zimbabwe (Makate, Wang, Makate, & Mango, 2016), the Philippines (Dressler *et al*., 2016) and India (Joshi, Gulati, & Birthal, 2007), which showed that both household wealth and perceived risk influence the land use decisions of smallholders. Instead of imposing technocratic solutions onto complex systems, land use strategies therefore need to match local conditions. Locally appropriate options, in turn, are best explored through the involvement of multiple stakeholders and sectors. An important caveat here is that some stakeholders may prefer land sharing because they perceive this to be a win‐win for food and biodiversity, when in fact, land sharing may not necessarily provide the best outcome for biodiversity conservation (Phalan *et al*., 2011). Moreover, since we included integrated land uses in both the forest and farmland in our definition of land sharing, stakeholders may have referred to either or both of these options in our interviews.

We also revealed a disparity between policy‐making and implementation‐level stakeholders, with a relatively greater preference for land sparing at policy‐making levels. This difference may be explained by the existing institutional context. Aspects of land sparing are enshrined in various formal institutions such as government policy, plans, and strategies (e.g., MoFED, 2010), whereas local institutions have traditionally favored land sharing. The notion of needing “more food for more people” – a common narrative in the natural sciences (Glamann, Hanspach, Abson, Collier, & Fischer, 2017) – dominates among policy‐making stakeholders. However, as recognized by local stakeholders, on the ground, food security is just as much about the accessibility and distribution to the target group (Fischer *et al*., 2014; Desquilbet *et al*., 2016). In line with our finding, studies in India (Rai & Bawa, 2013) and Madagascar (Pirard & Belna, 2012) indicated that policy stakeholders favor land sparing because it aligns with dominant development discourses. The singular focus on production, however, is usually caused by an inadequate understanding of the complex land use dynamics and challenges experienced by local people (Mertz & Mertens, 2017). The existing discourse thus causes two main misfits: (1) an incompatibility of policies with local conditions and preferences (Leventon & Antypas, 2012) and (2) various implementation deficits created through a gap between policy content and on‐ground capacities (Leventon & Antypas, 2012). In a landscape with multiple functions and multiple interests, the conflict of interest between stakeholders such as between the policy‐ and implementation‐level stakeholders could be reconciled though greater use of participatory processes (Groot, 2006). For instance, in Tanzania Hart *et al*. (2014) found that community participation enhanced sustainability, empowered community, and reconciled conflict among diverse stakeholders.

In contrast to the policy scale, the choice of land sharing is often favored in a context of local experience. For instance, an empirical study in the Philippines (Dressler *et al*., 2016) found that land sharing was supported by the local community, partly because it yielded sustainable outcomes in both social and ecological terms. Similarly, in Indonesia, Lee, Garcia‐Ulloa, Ghazoul, Obidzinski, and Koh (2014) indicated that land sharing was chosen by smallholders to improve their livelihoods. In addition to ecological justifications – as stipulated by the sparing‐sharing framework – social, institutional, and governance dimensions thus need to be integral parts of land use policy (Fischer *et al*., 2014; Kremen, 2015).

### 4.2 Capacity limitations

Implementation challenges related to stakeholder differences, biophysical conditions, and institutional factors. For example, community members may be reluctant to intensify, stakeholders’ interests may diverge, and different policies may be uncoordinated and incoherent. Existing work elsewhere suggests that such problems originate when policies are designed with minimal consideration of local context, community preferences, and capacities (Franzel & Houten, 1992); there is a lack of accommodation of diverse interests and goals (Veldhuizem *et al.*, 1997); and there is limited coordination and participation in designing, implementing, and enforcing policies (Hailemariam, 2004). To successfully design and implement suitable land use policies and strategies therefore requires the participation of a wide range of stakeholders, and needs to be compatible with the varied interests and local implementation capacities.

## 5 CONCLUSION

We reach three main conclusions. First, locally, the dichotomy between land sparing and sharing has limited value because existing patterns of land use are more heterogeneous. Second, agricultural landscapes are complex systems and involve stakeholders with multiple interests. The land sparing and sharing framework is grounded in ecological justifications, but on its own, does not account for social complexity. Next to ecological factors, social and institutional dimensions need to be considered in land use strategies if they are to sustainably harmonize food production and conservation goals. Third, there may be mismatches in understandings and strategic preferences between policy‐making stakeholders and formal institutions versus implementing stakeholders and informal institutions. To minimize such mismatches, land use policies should ensure stakeholder participation (both during policy design and implementation) and coordination between sectors (both at policy and implementation levels).

## Supporting information


**FIGURE S1** Map of the study area in southwest Ethiopia. Jimma zone, the study location, is indicated as the dark area on the Ethiopian map
**TABLE S1** Meaning of concepts as it is used in the article
**TABLE S2** A list of all stakeholders and their abbreviations.
