# Supplementary material for: The governance of land use strategies: Institutional and social dimensions of land sparing and land sharing
Source: Conserv Lett. 2017 Dec 13;11(3):e12429. doi: 10.1111/conl.12429 (PMC6049885; doi:10.1111/conl.12429)
Supplement: Supplementary file 1 — FIGURE S1 Map of the study area in southwest Ethiopia. Jimma zone, the study location, is indicated as the dark area on the Ethiopian map TABLE S1 Meaning of concepts as it is used in the article TABLE S2 A list of all stakeholders and their abbreviations. [file CONL-11-na-s001.pdf]

Supplementary Material

1. Study area

Ethiopia has a federal government consisting of nine regional states and two city administrations, which are demarcated on the basis of linguistics and ethnic lines. As stipulated in the Ethiopian constitution Article 46, sub-Article 2, states are delimited based on settlement pattern, linguistics, ethnic identity and the consent of the peoples concerned (FDRE, 1995). The administration of the country has five tiers: the national/federal level, regional states, zonal administration, district (hereafter woreda) administration and kebeles (the lowest formal administrative unit). Oromia region, home of the Oromo ethnic group, is the largest state in terms of population and area covered. Administratively, the region is classified into 18 zonal administrations. This study was conducted in the Jimma zone in Oromia regional state, southwestern Ethiopia (Fig. S1). The zone is located approximately 350 km southwest of the national and Oromia regional capital, Addis Ababa. Jimma zone constitutes 18 woredas and 513 kebeles. The total population of Jimma zone is estimated to be 3.14 million people (OBFED, 2012). Approximately 95% of the population of Jimma zone resides in rural areas (OBFED, 2012). Jimma zone is a center of origin for coffee (*Coffea arabica*). According to the Jimma Zone Bureau of Agriculture, Jimma zone accounts for 70% of the total coffee produced in the country (unpublished 2008 report).

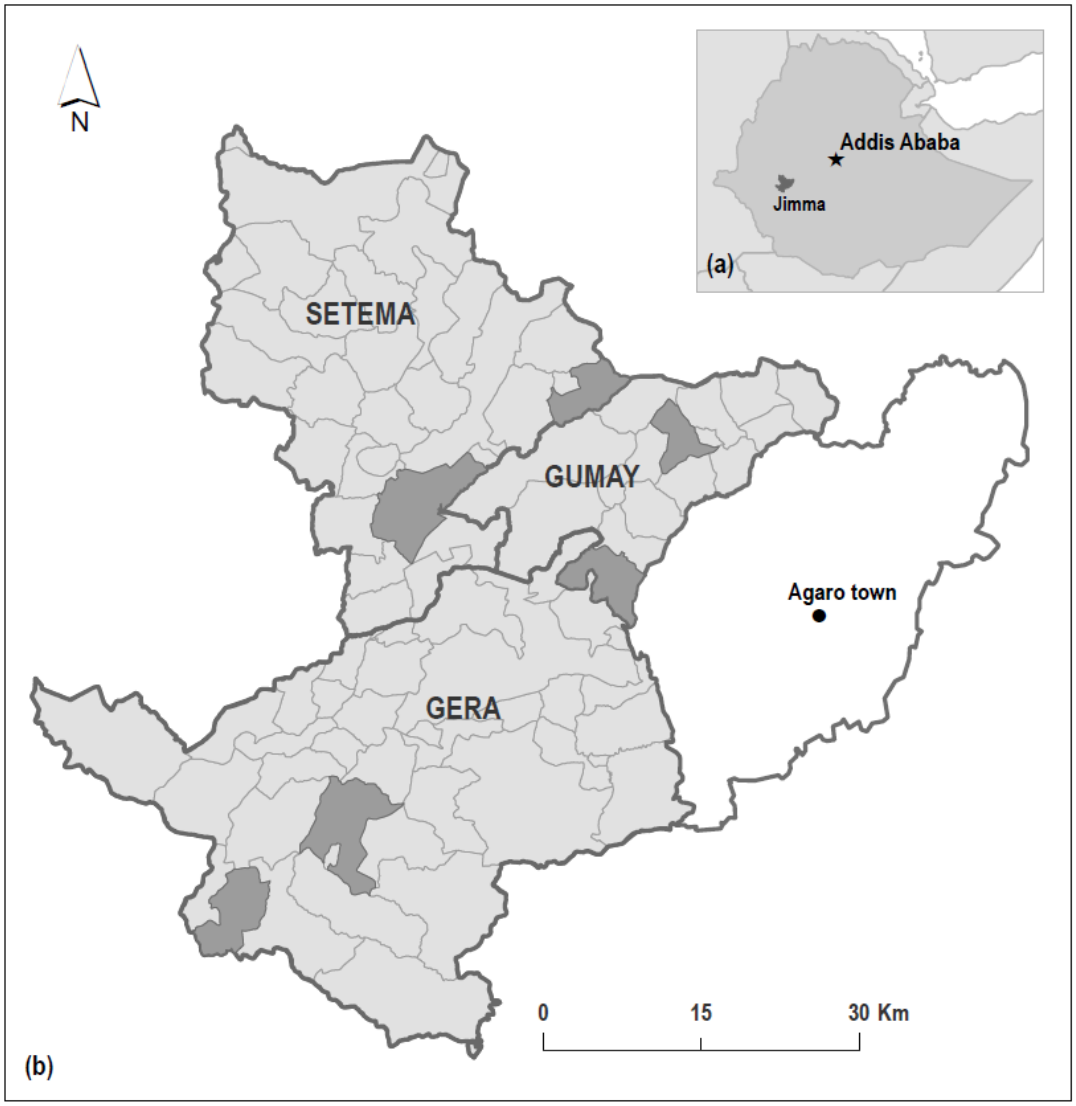

Figure S1. **(a)** Map of the study area in southwest Ethiopia. Jimma zone, the study location, is indicated as the dark area on the Ethiopian map. **(b)** The three study woredas Setema, Gumay and Gera. The six kebeles chosen for this study (Kuda Kufi, Berwerengo, Kela Hareri, Borcho Deka, Gido Bere, Difo Mani) are shaded. The focus group discussions and interviews with the community were conducted in these kebeles, which were purposively selected to cover a range of social and biophysical conditions within the study area.

2. Research Design

We selected our study area because it has rich but declining biodiversity (Ango et al. 2014). People in Jimma zone are relatively better off in terms of food security than in the drier parts of Ethiopia, but many inhabitants remain food insecure by international standards – seasonal food shortages, where meals need to be skipped or reduced, are common (CSA/WFP, 2014). Within Jimma zone, we focused on three woredas, namely Gumay, Gera, and Setema (see Fig. S1). Similarly, six kebeles (two in each woreda) were selected to cover gradients of forest cover, coffee production, and food security in the area. Therefore, for our governance of land use strategy analysis, we considered stakeholders from six kebeles, three woredas, as well as zonal, regional and national governance levels.

Stakeholders working on food security or biodiversity conservation (or both) were identified through bottom-up snowball sampling starting at the kebele (most local) level, to ensure that no important stakeholders were missed. First, kebele level stakeholders, including local community and on-ground development and conservation stakeholders, were identified through the help of local guides and administrators, to whom we had explained the scope and goal of the project. Accordingly, groups of farmers were identified and categorized into rich versus poor, drawing on taxation data from local government offices. The classification of wealth into two wealth classes was based on household assets such as land holdings, annual income and food security status. This categorization was used to explore differences in the preference of land use strategies between wealth categories. After this classification, key informant interviewees and focus group discussants were identified through the help of local guides – including kebele level agricultural development agents, health development agents, kebele leaders, and community group leaders. We used a set of pre-defined criteria in the selection of respondents to ensure both social as well as geographical

representativeness, and to minimize the potential bias caused due to social and geographic factors. Thus, we considered respondents' willingness and ability to discuss, and level of knowledge of food and biodiversity issues through their experience in the area. The level of formal education within the community was similar among wealthy and poor people, and we avoided the possibility of elite capture by separately interviewing different status groups and a diversity of respondents. For instance, within every kebele, there were three community groups composed of inhabitants who were clustered based on their geographical settlement in the kebele.

In both focus group discussions and key informant interviews, all kebele level stakeholders (community as well as other governmental and non-governmental organizations) were asked about five general themes: (1) General background and trends in land use in the area; (2) land use preferences; (3) justification for the preference; (4) challenges for the implementation of the preferred land use system; and (5) other stakeholders involved in the governance of food security and biodiversity, both horizontally (i.e. within the kebele) and vertically (i.e. at higher governance levels).

Drawing on information gathered from the fifth question listed above, we considered all stakeholders involved in the production and supply, access, utilization and agency dimensions of food security, as well as farm and forest dimensions of biodiversity management (see Table S1 for explanations of concepts). Based on this process at the kebele level, we identified woreda level stakeholders, and continued this process up to the national level, until no new stakeholders were mentioned. This process of stakeholder identification generated 244 stakeholders in the governance of food security and biodiversity from local up to the national/federal level. However, because food security and biodiversity governance are broader concepts than just land governance, only 80 of the 244 stakeholders were directly involved in the decision related to land use. Some of

the stakeholders, for instance credit and finance associations (OCSA) and youth and sports office (YOSP), were part of food security governance but were not involved in land use decisions. Thus, we considered only those 80 stakeholders directly involved with land governance in this study (Table S2). We administered interviews with stakeholders through their respective representatives, which included heads or deputies of the organization, planning officers, and senior personnel.

The process of data collection took two steps. First, we pre-tested the data collection tools in August 2015 to see whether the prepared protocol would be properly understood and generate the intended data. We then modified the tools accordingly based on the field trial. Second, we conducted the actual data collection between October-February 2015-2016. Because the terminology and concept of "land sparing" versus "land sharing" was unknown to stakeholders, we explained these concepts to all stakeholders before we commenced the interview. We described land sparing as a strategy that is a spatial segregation of agricultural land and biodiversity conservation areas whereas land sharing was described as a strategy that attempts to integrate conservation and production on the same land (see Table S1 for details). We audio recorded and took notes of all the interviews and discussions after obtaining voluntary, informed consent by the stakeholders.

For analysis, we translated and transcribed all the 80 recordings and field notes separately for each of the stakeholders. Following this, we used NVivo software version 11 to code and analyze the data. In NVivo, we deductively created three separate nodes for land sparing, land sharing and mixed strategies; and classified stakeholders according to their preferences of sparing, sharing or a mix; and identified their responsibilities in policy-making versus implementation. We then inductively created sub-nodes under each of the categories and coded arguments or justifications provided by the stakeholders for their preferred land use strategy. Similarly, we created sub-nodes

for the capacity limitations for each of the three categories and coded stakeholder's response. Finally, the coded data were categorized and themes emerging were analyzed using content analysis.

3. Concepts used in the paper

Table S1: Meaning of concepts as it is used in the paper

**Concept**	**Description**
Food security	Food security is a broad concept that has multiple definitions (see Maxwell and Smith, et al. 1992). Here, we adopted the definition provided by world food program: “Food security exists when all people have physical, social and economic access to sufficient, safe, nutritious and preferred food at all times, such that they can lead a healthy and productive life” (FAO, 2014). This conceptualization of food security entails four major dimensions of food security: 1) Availability/ production dimension: this dimension involves ensuring that food is sufficiently available to all people at all times. Accordingly, stakeholders involved in the food production sector were considered in our assessment of land use preference; 2) Economic and physical access: this dimension comprises ensuring that all people have the physical and financial capacity to afford nutritious and preferred food. Thus, stakeholders mandated with financial and capacity empowerment of the community were considered in scoping this study; 3) Utilization dimension: this dimension focuses on the adequacy and nutritional values of food consumed and hence involves stakeholders from health and other dietary service providers whom were also part of this study. 4) Stability dimension: this component of food security is concerned with the uninterrupted functioning of the above dimensions, and hence involves institutions such as administration,

	regulatory and monitoring agencies. These stakeholders were also part of this study. Thus, at first, all stakeholders involved in these dimensions were considered. From these stakeholders, however, those who were directly related with the land use governance were considered in the interviews and focus group discussions that were the specific purpose of this paper.
Biodiversity	Biodiversity is another broad concept used in this paper. For this paper, we adopted the definition of biodiversity as provided by Convention on Biological Diversity (CBD, 1992) which stated biodiversity as: “the variability among living organisms from all sources including inter alia, terrestrial, marine and other aquatic ecosystems and the ecological complexes of which they are part; this includes diversity within species, between species, and of ecosystems” (CBD, 1992). We considered all stakeholders involved in the governance of biodiversity for both farmland and forest land. After this broad framing, we identified those stakeholders who had a direct stake in land governance.
Land sparing	Land sparing describes a spatial segregation of areas used for intensive farming and areas strictly protected for biodiversity conservation. It is a land use strategy that supports the segregation and strict conservation of biodiversity through creation of protected areas along with agricultural land intensification through extensive use of external inputs such as agrochemicals to compensate the land spared for biodiversity conservation.
Land sharing	Broadly, land sharing indicates a strategy that combines food production and biodiversity conservation on the same land thus providing lower levels of protection but also lower amounts of external inputs. Land sharing is conceptualized in

	different ways based on the context. For instance, it could mean using agricultural practices that support biodiverse and heterogeneous agricultural systems that may or may not include forest fragments. It could also mean retaining forest in the traditional agricultural land use system. The proxy used in framing land sharing varies mainly depending on agricultural yield level, agricultural practices or heterogeneity of agricultural landscape (see Kremen, 2015). To avoid the ambiguity associated with the concept, we made explicit to all stakeholders that land sharing involves the two conditions of traditional low external input agricultural farming with farm heterogeneity. This could happen both on farmland as well as on forest land. For instance, producing coffee in the forest is a common practice of the landscape in the study area. Similarly, trees on farmland as patches or scattered trees are common in the landscape. Hence, in our case, we conceptualized land sharing as a practice of maintaining trees on farmland with low agricultural intensification and producing coffee in the shade of forest land.
Mixed land use system	A mixed land use strategy combines elements of both the land sharing and land sparing strategies in a mosaic of different land use types. The concept is similar with what Kremen (2015) emphasized in her paper as “Both-and” type of land use policy options. We considered a mixed land use system when the stakeholders preferred to see both land sharing and land sparing on the same land use system. For instance, some stakeholders preferred the use of external inputs such as agrochemicals while maintaining trees and patches of forest on the farm land, or the use of traditional agricultural farming with less applications of agrochemicals on the farm land, and still sparing the conservation land as a protected area.

Agricultural intensification	We considered this to be an agricultural practice to raise yield output per unit land area. The increase in yield per unit area could be achieved either through *conventional intensification* which support the intensive use of irrigation and agrochemicals, high-yielding crop, and farm mechanization. An alternative type of intensification is *agro-ecological intensification* which supports agricultural yield increase through natural means such as using agroforestry techniques (see Loos et al. 2014)

4. List of stakeholders

Table S2. A list of all stakeholders and their abbreviations. The first column gives the stakeholder's acronym while the full name of the actor is given in the second column. The third and fourth columns indicate stakeholders' administrative levels and the name of the corresponding administrative level. The fifth column shows the type of organization including CG (community groups); GO (governmental organizations); FR (farmers); CA (semi-autonomous cooperative agency) and NGO (non-governmental organizations). The last column indicates the gender characteristics of participants as M (male respondents or discussants) and F (female respondents or discussants).

Actors acronym	Full name of stakeholders	Administrative level	Administrative name	Type of organization	Gender	
					M	F
PoK1	Poor community group	Kebele	Kuda Kufi (KK)	CG	4	3
PoK2	Poor community representative	Kebele	Kuda Kufi (KK)	FR		1
RiK1	Rich community groups	Kebele	Kuda Kufi (KK)	CG	3	3
RiK2	Rich community respondent	Kebele	Kuda Kufi (KK)	FR	1	
GeK	General community	Kebele	Kuda Kufi (KK)	CG	4	3
NeK	Community network leaders	Kebele	Kuda Kufi (KK)	CG	3	
LeK	Kebele leaders	Kebele	Kuda Kufi (KK)	GO	1	
CoPK	Jawi multipurpose cooperative	Kebele	Kuda Kufi (KK)	CG	1	
PoB1	Poor community group	Kebele	Kuda Kufi (KK)	CG	2	4

PoB2	Poor community representative	Kebele	Kuda Kufi (KK)	FR		1
RiB	Rich community groups	Kebele	Berwerengo (BW)	CG	5	
RiB2	Rich community representative	Kebele	Berwerengo (BW)	FR	1	
GeB	General community	Kebele	Berwerengo (BW)	CG	3	2
NeB	Community network leaders	Kebele	Berwerengo (BW)	CG	3	
PoD1	Poor community group	Kebele	Difo Mani (DM)	CG	4	4
PoD2	Poor community representative	Kebele	Difo Mani (DM)	FR	1	
RiD1	Rich community group	Kebele	Difo Mani (DM)	CG	5	2
RiD2	Rich community representative	Kebele	Difo Mani (DM)	FR	1	
GeD	General community	Kebele	Difo Mani (DM)	CG	3	2
HeD	Health extension office	Kebele	Difo Mani (DM)	GO		1
DaD	Development agent/agricultural extension office	Kebele	Difo Mani (DM)	GO	1	
PoG	Poor community group	Kebele	Gido Bere (GB)	CG	3	2
RiG	Rich community group	Kebele	Gido Bere (GB)	CG	2	2
RiG2	Rich community representative	Kebele	Gido Bere (GB)	FR	1	
GeG	General community	Kebele	Gido Bere (GB)	CG	5	2
DaG	Development agent/agricultural extension office	Kebele	Gido Bere (GB)	GO	2	
PoK1	Poor community group	Kebele	Kela Hareri (KH)	CG	3	3
PoK2	Poor community representative	Kebele	Kela Hareri (KH)	FR	1	
Rik1	Rich community group	Kebele	Kela Hareri (KH)	CG	3	3
Rik2	Rich community representative	Kebele	Kela Hareri (KH)	FR	1	
GeK1	General community	Kebele	Kela Hareri (KH)	CG	5	2

NeK1	Community network leaders	Kebele	Kela Hareri (KH)	CG	3	
LeK1	Kebele leaders	Kebele	Kela Hareri (KH)	GO	1	
PoB1	Poor community group	Kebele	Borcho Deka (BD)	CG	4	3
PoB2	Poor community representative	Kebele	Borcho Deka (BD)	FR		1
RiB1	Rich community group	Kebele	Borcho Deka (BD)	CG	3	3
RiB2	Rich community representative	Kebele	Borcho Deka (BD)	FR	1	
GeB1	General community	Kebele	Borcho Deka (BD)	CG	6	3
LeB1	Kebele leaders	Kebele	Borcho Deka (BD)	GO	1	
DaB1	Development agent/agricultural extension office	Kebele	Borcho Deka (BD)	GO	1	
BOAGU	Bureau of agriculture and natural resources office	Woreda	Gumay (GM)	GO	1	
LAEMGU	Land administration and environmental management	Woreda	Gumay (GM)	GO	1	
IRRGU	Irrigation development authority office	Woreda	Gumay (GM)	GO	1	
DPPGU	Disaster prevention and preparedness office	Woreda	Gumay (GM)	GO	1	
COPGU	Cooperative development office	Woreda	Gumay (GM)	GO	1	
OFWEGU	Oromia forest and wildlife enterprise office	Woreda	Gumay (GM)	GO	1	
TAMDGU	Trade and market development office	Woreda	Gumay (GM)	GO	1	
BOAGE	Bureau of agriculture and natural resources	Woreda	Gera (GE)	GO	1	
IRRGE	Irrigation development authority office	Woreda	Gera (GE)	GO	1	
LIVGE	Livestock and fisheries development and marketing	Woreda	Gera (GE)	GO	1	
COPGE	Cooperative development office	Woreda	Gera (GE)	GO		1

LAEMGE	Land administration and environmental management	Woreda	Gera (GE)	GO	1	
MEIGE	Micro finance enterprise office	Woreda	Gera (GE)	GO	1	
BOASE	Bureau of agriculture and natural resources	Woreda	Setema (SE)	GO	1	
LAEMSE	Land administration and environmental management	Woreda	Setema (SE)	GO	1	
IRRSE	Irrigation development authority office	Woreda	Setema (SE)	GO	1	
LIVSE	Livestock and fisheries development and marketing	Woreda	Setema (SE)	GO	1	
COPSE	Cooperative development office	Woreda	Setema (SE)	GO		1
TAMDSE	Trade and market development office	Woreda	Setema (SE)	GO	1	
DPPSE	Disaster prevention and preparedness office	Woreda	Setema (SE)	GO	1	
OFWESE	Oromia forest and wildlife enterprise office	Woreda	Setema (SE)	GO	1	
BOAJZ	Bureau of agriculture and natural resources	Zone	Jimma (JI)	GO	1	
IRRJZ	Irrigation development authority office	Zone	Jimma (JI)	GO	1	
LAEMJZ	Land administration and environmental management	Zone	Jimma (JI)	GO	1	
CASCAJZ	Capacity building for scaling up best practices project	Zone	Jimma (JI)	NGO	3	
EARIJZ	Ethiopian agricultural research institute	Zone	Jimma (JI)	GO	1	1
AMEJZ	Agricultural mechanization research center	Zone	Jimma (JI)	GO	1	
OFWEJZ	Oromia forest and wildlife enterprise	Zone	Jimma (JI)	GO	1	

IBC	Institute of biodiversity conservation	Zone	Jimma (JI)	GO	1	1
AGPJZ	Agricultural growth program office	Zone	Jimma (JI)	NGO	1	
IRROR	Irrigation development authority office	Region	Oromia (OR)	GO	1	
COPOR	Cooperative development office	Region	Oromia (OR)	GO	1	
DPPCOR	Disaster prevention and preparedness office	Region	Oromia (OR)	GO	1	
OCA	Oromia cooperative agency office	Region	Oromia (OR)	CA	1	
BOA	Bureau of agriculture and natural resources	Region	Oromia (OR)	GO	1	
MOA	Ministry of agriculture and natural resources	Federal	Ethiopia (ET)	GO	1	
MOL	Ministry of livestock development and fisheries	Federal	Ethiopia (ET)	GO	1	
IBD	Ethiopian biodiversity institute	Federal	Ethiopia (ET)	GO	1	
MOFECC	Ministry of environment, forest and climate change	Federal	Ethiopia (ET)	GO	1	
EWCA	Ethiopian wildlife conservation authority	Federal	Ethiopia (ET)	GO	1	

References

Ango, T. G., Börjeson, L., Senbeta, F., & Hylander, K. (2014). Balancing ecosystem services and disservices: Smallholder farmers' use and management of forest and trees in an agricultural landscape in southwestern Ethiopia. Ecology and Society, 19(1), 30. https://doi.org/10.5751/es-06279-190130

CBD (Convention on Biological Diversity) (1992). Convention on Biological Diversity. Montreal, Canada: Secretariat of the Convention on Biological Diversity.

Constitution of the Federal Democratic Republic of Ethiopia, 21 August (1995). Available at: http://www.refworld.org/docid/3ae6b5a84.html [accessed 1 October 2017]

CSA/WFP (Ethiopian Central Statistical Agency/World Food Programme) (2014). Ethiopia: Comprehensive food security and vulnerability analysis, 2014. Addis Ababa, Ethiopia: Physical Planning Department.

FAO (Food and Agriculture Organization of the United Nations). 2014. The state of food insecurity in the world. Rome, Italy: Food and Agriculture Organization of the United Nations.

Jimma Zone Bureau of Agriculture, (2008). Annual socioeconomic condition report of Jimma zone (unpublished 2008 report).

Kremen, C. (2015). Reframing the land-sparing/land-sharing debate for biodiversity conservation. Annals of the New York Academy of Sciences, 1355(1), 52–76.

Loos, J., Abson, D. J., Chappell, M. J., Hanspach, J., Mikulcak, F., Tichit, M., & Fischer, J. (2014). Putting meaning back into "sustainable intensification". Frontier in Ecology and the Environment,12(6), 356–361, https://doi.org/10.1890/130157

Maxwell, S., & Smith, M. (1992). Household food security: a conceptual review. In: Maxwell, S., & Frankenberger, T. R. (Eds). Household food security: concepts, indicators, measurements – A technical review. New York and Rome: UNICEF/IFAD.

OBFED (Oromia Bureau of Finance and Economic Development) (2012). Condensed Physical Geography of Oromiya. Addis Ababa, Ethiopia: Physical Planning Department.
